# COVID-19 Outbreak during Summer Courses at an Elementary School

**DOI:** 10.3390/children10030418

**Published:** 2023-02-22

**Authors:** Carlos Pantoja-Meléndez, Guadalupe García-De la Torre, Mónica Duran-Robertson, Kenneth Peterson-Marquard, Silvia Núñez-Amador, Víctor Gomez-Bocanegra, Gabriela Ibáñez-Cervantes, Cruz Vargas-De-León, Mónica Cureño-Diaz

**Affiliations:** 1 Departamento de Salud Pública, Facultad de Medicina, Universidad Nacional Autónoma de México, Ciudad de México 04510, Mexico; 2 Peterson Schools, Ciudad de México 05249, Mexico; 3 Secretaria de Marina, Ciudad de México 04470, Mexico; 4 División de Investigación, Hospital Juárez de México, Ciudad de México 07760, Mexico; 5 Sección de Estudios de Posgrado, Escuela Superior de Medicina, Instituto Politécnico Nacional, Ciudad de México 11340, Mexico; 6 Dirección de Investigación y Enseñanza, Hospital Juárez de México, Ciudad de México 07760, Mexico

**Keywords:** SARS-CoV-2, school outbreak, case and contact research, attack rate, containment

## Abstract

Due to the COVID-19 emergency, face-to-face classes were suspended. After the vaccination of teachers and to mitigate educational backwardness, the schools have begun to reopen with protocols established by the government. Here, we investigated the COVID-19 outbreak in summer courses during the reopening of a private elementary school in July 2021. We report confirmed cases of COVID-19 in staff members, students, and their families. A total community of 290 people was part of this study, and we built the contact network. The clinical features of all cases are described. We used the methodology of cases and contacts. The index case was identified by epidemiological tracking, and containment measures were activated, as well as further infection chains in the setting. We estimate the attack rate for staff members at 15.68% (95% CI 7.0–28.6), students at 12.24% (95% CI 4.6–24.8), and family members at 2.6% (95% CI 0.8–6.0). An incubation period of 48–72 h was determined. A student–teacher–student–family transmission sequence was identified. The area where the infection was identified was the school swimming pool, an area where face masks are not worn or, in some cases, inadequately used. Finally, we continue with intermittent staff testing and early detection actions, reinforcing prevention measures, environmental control, cleaning, and educational interventions with students regarding the implementation of preventive measures through classes led by school health staff.

## 1. Introduction

Since the appearance of the SARS-CoV-2 coronavirus at the end of 2019, several forms of social organizations have been put to the test. Once the pandemic had been declared by the World Health Organization (WHO) in each country, the intermittent use of social demobilization measures was necessary, according to their respective internal policies. One of these policies is the cancellation of face-to-face classes, though some countries opted for mixed face-to-face and distance education techniques or intermittent closures depending on the dynamics of local contagion.

Reopening colleges and universities during the COVID-19 pandemic poses a special challenge worldwide. One of the few countries where schools operated normally was Taiwan. A combination of containment (access control with contact tracing and quarantine) and mitigation (hygiene, sanitation, ventilation, and social distance) procedures may make it possible, according to Taiwan’s experience, to safely reopen colleges and universities this fall [1]. On 17 May 2020, all school classes in Israel reopened with the requirement for daily health reports, hygiene, facemasks, social distancing, and minimal interaction between classes [2]. Ezeonu, Uneke, and Ezeonu [3] conducted a rapid review from March 2020 to August 2020 of the strategies adopted in the reopening of schools in China, Taiwan, South Korea, Norway, Denmark, Germany, Australia, and Israel. All these countries started with phased reopening and a reduction in class size. In other countries, masks had to be worn. A stringent cleaning regimen was followed for high-touch surfaces and hand hygiene [3]. In particular, elementary schools [4], secondary schools [5], high schools [2,6,7], and boys’ overnight summer school retreats [8] were reopened. The description of the outbreak, the epidemiological investigation, and the attack rates on staff members and students were documented. 

In Mexico, face-to-face classes were suspended on 23 March 2020 in preschools, primary and secondary schools, as well as upper middle and higher schools, dependent on the Ministry of Public Education (Spanish acronym: SEP), as a preventive measure to reduce the spread of COVID-19 impact in the national territory [9]. 

The National Institute of Statistics and Geography Health (Spanish acronym: INEGI) conducted the Survey to Measure the COVID-19 Impact on Education (Spanish acronym: ECOVID-ED) due to the provisional cancellation of face-to-face classes for students from 3 to 29 years of age in school cycles 2019–2020 and 2020–2021. According to this survey, 2.0% of students in public schools and 4.2% in private schools did not complete the school cycles 2019–2020, and around 812,000 children and adolescents between the ages of 3 and 12 did not re-enroll in the 2020–2021 school cycles for reasons related to the pandemic [10]. A lag in the country is also estimated at the level of early childhood development due to the closure of physical spaces for programs and activities of initial and preschool education [11]. On 7 June 2021, UNICEF urged Mexico to return to schools in person as a key to the continuity of education and recovery of learning that will help mitigate problems faced by an entire generation resulting from school closures [11]. 

In May 2020, the Ministry of Health and the Ministry of Public Education jointly prepared the Guide for the Responsible and Orderly Return to Schools for the 2021–2022 school year, and updated it in August 2021 with version 3.0, to establish protocols to guarantee that children and adolescents return to safe educational spaces, free of risk for infection [12,13]. 

On 16 April 2021, the Government of Mexico began the vaccination strategy for teachers [13], which would allow the reopening of schools. In the private elementary schools—the subject of this study—educational activities with the students were resumed before the end of the 2020–2021 school cycle and progressed immediately into summer courses, which included a greater number of physical activities. In our study, we investigated the COVID-19 outbreak in summer courses during the reopening of a private elementary school in July 2021.

## 2. Materials and Methods

### 2.1. Characteristics of the School Population

The outbreak location was a private elementary school in Álvaro Obregón borough, Mexico City, which serves 49 students in grades K1 (3 years old), K2 (4 years old), K3 (5 years old), PF (6 years old), 1° (7 years old) and has 51 staff members. Each grade includes 2 to 13 students in single classrooms. Of the staff members who were confirmed cases, seven of the eight completed the vaccination schedule by viral vector-based vaccines (6 CanSino and 1 Aztra-Zeneca) in May 2022. In the case of the students, none were vaccinated according to current regulations [9,12,13]. 

**Figure 1:**
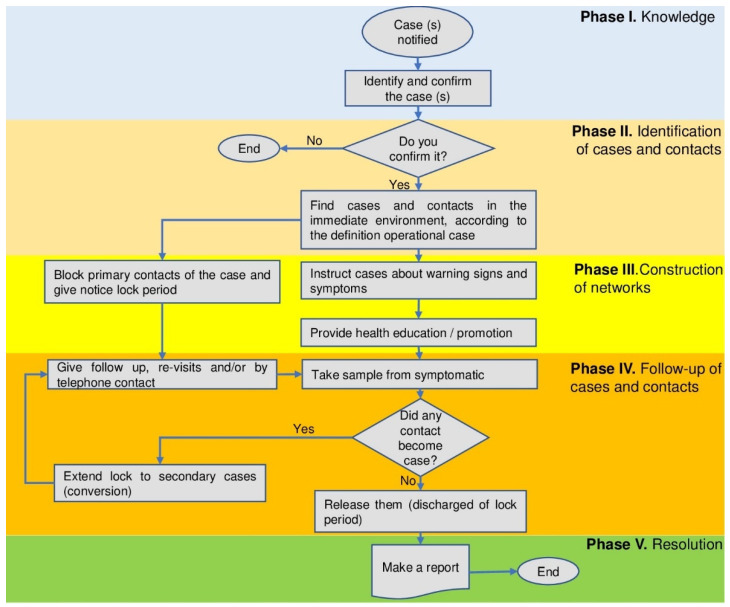
Flowchart of the case–contact study.

In this study, we use the methodology of cases and contacts, which is summarized in Figure 1.

### 2.2. Prevention and Control Protocol for COVID-19 Virus

The elementary school implemented the measures of the local health authority: 1. cleaning and disinfecting areas, guided by colors to avoid cross-contamination; 2. frequency of cleaning based on the intensity of use of the areas; 3. use of air extraction systems in classrooms that guarantee at least 10 air exchanges per hour; 4. average CO_2_ measurements of 550 ppm or less; 5. mandatory use of face masks; 6. monitoring and ensuring that the use of water inside the facilities is within chlorine standards [9]. 

In addition, a self-diagnosis by students and parents with an app to determine daily risk factors, signs, or symptoms, verified before entering the facilities, was implemented. Moreover, clinical examinations were conducted for teaching and administrative staff and students with risk factors, and randomly with 10% of the population daily. Specific follow-up was completed with students who did not attend school to determine the cause of their absence. The strengthening and implementation of self-care education actions and care and hygiene measures for students were taught by school health personnel.

### 2.3. Beginning of the Outbreak in Summer Courses

On 5 July 2021, COVID-19 screening tests were carried out on all staff members as a requirement to start the summer courses. The network of contacts under investigation involved a primary case of one student (K3P) at the school. A parent reported during the interview that her daughter’s nanny (FP) had symptoms of COVID-19, which was confirmed on 8 July. In turn, K3P was confirmed on 10 July. K3P had close contact with staff members on 7 July.

### 2.4. Clinical Examinations and Treatments

A staff member, as part of the prevention and control protocol of the school, reported to the paramedical area with the presentation of respiratory signs and symptoms (runny nose and cough). 

Students with respiratory symptoms were referred and reviewed by their pediatricians, who indicated a SARS-CoV-2 real-time polymerase-mediated chain reaction preceded by a reverse transcription (RT-qPCR) test of a nasopharyngeal swab sample. RT-qPCR was performed using specific primers and probes with the Super-Script III Platinum One-step qT-qPCR Kit [14]. Symptomatic treatments were implemented for staff members. In addition, data on demographics and disease history were registered.

### 2.5. Epidemiology Investigation

An extensive follow-up of cases was carried out. Screening antigen tests [15] were carried out on all staff members on 13 July 2021 before starting the summer courses. Five staff members who had contact with the primary case were examined. When staff members presented a positive screening antigen test, they underwent an RT-qPCR test. 

The staff was notified of their test results, and isolation was indicated, as well as that of their primary contacts (family, work, or cohabitation), for 14 days, in accordance with current regulations [9]. At the same time, they were informed of warning signs and symptoms. During the 14-day isolation period, everyone was asked to report their symptoms, including rhinorrhea, fever, cough, anosmia, dysgeusia, myalgia, etc. 

Since the primary contacts of the cases (staff) tested negative, they were not asked to isolate, but were followed up with for the next 10 days, with no change in them, their family members, or other primary contacts. The students in the classroom where cases occurred were also not isolated but were followed up with.

### 2.6. Outbreak Definition

The presentation of two or more instances that are linked in terms of time, location, and individual is referred to be an outbreak. A single case under surveillance in a region where the illness was unknown is also regarded as an outbreak.

### 2.7. Case Definition

Confirmed cases were those who tested positive for COVID-19 using an RT-qPCR test. A suspected case was defined as having an exposure history of COVID-19 and two or more symptoms of COVID-19. The incubation period was defined as the length of time between the earliest date of contact with a source of transmission and symptoms experienced. The index was defined as the first documented subject in the school outbreak. The primary case was defined as the case that started the outbreak.

### 2.8. Contacts Definition

We defined close contacts as individuals who had close (less than 1.5 m away) and prolonged (more than 15 min of contact time) encounters. All other contacts were defined as casual contacts.

### 2.9. Construction of Contact Networks

The contact networks were built with four key elements: nodes with white fill that represent the role within the school and their family, nodes with yellow fill that represent confirmed cases, directional edges in black that represent an interaction between two people, and directional edges in green that represent a possible family contagion. 

We build the contact networks in the following three steps. 

First, we denote staff members as follows: A is an administrative staff, D is a teacher, I is an intendancy, L is a cleaning staff, and V is a surveillance staff. We denote the students as follows: CB is a children’s community, K1 is a kinder 1, K2 is a kinder 2, K3 is a kinder 3, PF is a pre-first grade, and 1° is a first grade. The family is denoted with F. 

Second, the directional edges in black established the interaction between staff members, interaction between staff members and students, interaction between staff members and their families, and interaction between students and their families. 

Finally, using the closed-circuit monitoring system, the videos of the days of possible exposure of the confirmed cases were reviewed. We denoted the family contact with directional edges in green. Containment measures, such as the isolation of the subject and their families, are denoted by a red cluster.

### 2.10. Statistical Analysis

Descriptive analyses were performed to estimate the attack rate of COVID-19 and the exact confidence interval in staff members, students, families, and the community. We calculate the reproductive number ($R_{0}$) using the following formula $R_{0}=log\;\left(\frac{S_{0}}{1-AR}\right)/\left(AR-\left(1-S_{0}\right)\right)$ proposed by Dietz [16], where AR is the attack rate and $S_{0}$ is the initial percentage of the susceptible population. Counts and percentages were used to summarize the signs and symptoms of patients with COVID-19. The Fisher’s exact test was used to compare the signs and symptoms between staff members and students. *p*-values < 0.05 were considered statistically significant. Analyses were performed with R software, version 4.1.3.

### 2.11. Ethical Considerations

This study was approved by the research ethics committee of the Hospital Juarez de Mexico (HJM). The present study complied with the basic principles of human research following the Declaration of Helsinki of the Medical Association. Personal data of the subjects that compromise their privacy are not shown. The school, study participants, and school authorities agreed to publish data from the outbreak.

## 3. Results

### 3.1. Description of the Outbreak and Contact Networks

As of 5 July 2021, all staff started with a negative test (time zero), making it possible to delimit the period of exposure. A parent reported during the interview that her daughter’s nanny (FP) had symptoms of COVID-19, which had been confirmed by an RT-qPCR test on 8 July. This student (K3P) was confirmed by an RT-qPCR test on 10 July as positive for COVID-19. K3P had direct contact with four teachers and an activities coordinator since 7 July in the pool area, where students do not wear face masks, and teachers use a face shield as a barrier mechanism. 

On 12 July 2021, a staff member (A1), as part of the prevention and control protocol of one of the schools, reported to the paramedical area with the presentation of respiratory signs and symptoms (runny nose and cough). Immediately after this suspicious case, outbreak containment activities were initiated, and students were clinically evaluated, in addition to calling all staff the next day for rapid and RT-qPCR testing. 

On 13 July, antigen tests were performed on all staff for immediate diagnostic screening. A total of 48 antigen tests were performed. Three of the teachers started with mild symptoms on 9 July (D1, D2, and A1), attributing their symptoms to the change in temperature in Mexico City, though they were confirmed for COVID-19 during screening on 13 July, which was 100% corroborated by the RT-qPCR test. The other two teachers who had contact with K3P tested negative and never had symptoms. 

In the clinical evaluation of the students on 13 July 2021, eight cases with respiratory symptoms were identified. These were referred and reviewed by their pediatricians, who indicated tests for COVID-19, finding four positive cases with the RT-qPCR test. Upon testing their siblings, one more case was detected, which was asymptomatic. The parents of the children were asked to isolate their primary contacts, including themselves, for 10 days. Four of the eight children with symptoms tested negative with RT-qPCR. 

The first three cases presented contact with K3P, so it was defined as the “primary case”, with the latter not being the index case.

The first case, or “index case” (A1), was identified on 12 July, having started with symptoms on 9 July. The exposure period and the occurrence of cases within the incubation period of 48–72 h were determined. 

Among the first three symptomatic cases, one of the teachers (D2) has a direct family relationship (mother–daughter) with Administrative 2 (A2), who developed symptoms 48 h–72 h after the onset of symptoms in case D2. Cases D3 and D4 presented symptoms 72 h (12 July) after contact with A1 and D1, although the test was confirmed on 12 July. Cases D5 and D6 were not symptomatic on 13 July; however, D6 started symptomatology on 14 July. 

Isolation was recommended not only to positive cases, but also to primary contacts (work, family, or cohabitation) on 13 and 14 July, presenting eight cases, all in-family (primary contacts), approximately 72 h later, with the last case reported on 18 July. 

**Figure 2:**
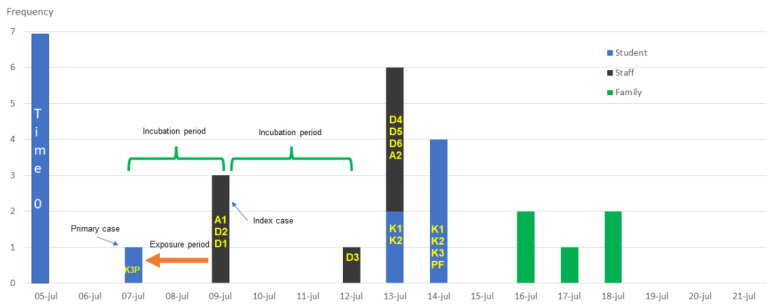
Epidemic curve of all cases with SARS-CoV-2 infection in the elementary school population. All cases with SARS-CoV-2 infection are plotted by the timeline of the confirmed case presentation.

In Figure 2, we show the epidemic curve that describes the timeline of the confirmed case presentation.

The staff and students were followed up with, as well as their primary contacts, and no more cases were found in the following 30 days. 

**Figure 3:**
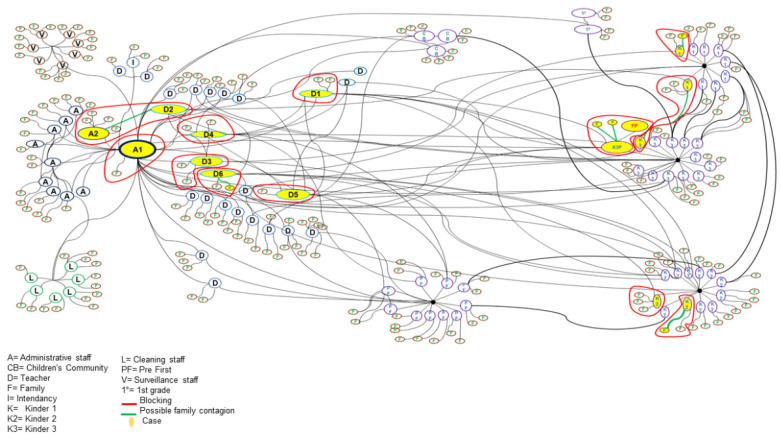
Network diagram of contagions and blocks.

It was possible to identify a student–teacher–student–family transmission sequence. It was also possible to clearly identify an outbreak of what is called a disseminated source. In Figure 3, we illustrate the networks of contacts. 

As part of the current regulations, as of 14 July 2021 (once RT-qPCR results became available), cases and outbreaks were reported to local health authorities.

### 3.2. Attack Rate and Reproductive Number at School

The initial attack rate was 5.88%, and the attack rate for associated primary and subsequent secondary contacts was 5.57%. The attack rate for staff was calculated at 15.68% (95% CI 7.0–28.6); for students, it was 12.24% (95% CI 4.6–24.8); and for family members, it was 2.6% (95% CI 0.8–6.0); resulting in a community or global attack rate of 6.6% (95% CI 3.9–10.0). The FP nanny was not considered a family case. Although methodologically, it is the source of the outbreak, it was not considered for the purpose of analyzing the information due to the difficulty in establishing the chains of contagion (Table 1).

The reproductive number for staff was calculated at 3.40, and for staff and students, it was 4.04.

### 3.3. Clinical Features of COVID-19 Subjects

In the evolution of the adult cases (personal), we present two outstanding cases: A1 and D1. The index case (A1) is a 33-year-old male. He presented with respiratory distress, pneumonia, general malaise, and hair loss. He needed oxygen therapy for recovery, which was achieved approximately 25 days after the onset of signs and symptoms, and presently continuing in respiratory rehabilitation. It is worth mentioning that this case is a person without comorbidities and was vaccinated with CanSino in May 2021. Case D1 was a 37-year-old female. She had runny nose, cough, anosmia, dysgeusia, and myalgia, and no comorbidities. She was vaccinated with CanSino in May 2021. In February 2021, she presented signs and symptoms of COVID-19 that were confirmed by an RT-qPCR test. 

The most frequent sign in the students was rhinorrhea, followed by fever. None of the children were vaccinated according to local regulations. Two of the students were asymptomatic. Regarding the staff, seven of them presented a clinical picture, with rhinorrhea and cough as the main signs and only one asymptomatic case. The difference in frequencies of cough (*p* = 0.005), anosmia (*p* = 0.031), and dysgeusia (*p* = 0.031) between the positive staff group and positive student group was statistically significant (Table 2).

### 3.4. Vaccination of Staff Members

In May 2021, all staff working in the school (surveillance, teachers, administrative, intendancy, and cleaning) were vaccinated by the Secretaries of Health and Education of Mexico. None of the students were vaccinated at the time. At the school, the staff was administered the following adenovirus vector-based vaccines against SARS-CoV-2 infection: AstraZeneca and CanSino. Thirty-nine members were administered the complete scheme with the CanSino vaccine (single dose). Eight other staff members were vaccinated with Astra-Zeneca (complete schedule with two doses). Staff who recently started working at the school were not vaccinated since they were not previously registered for the national vaccination of education staff. Table 3 shows the distribution of vaccinated and unvaccinated personnel versus COVID-19 vaccines.

From Table 3, we can see that there was only one infected case of the four unvaccinated (25.0%) and seven infected cases of the 47 vaccinated (14.9%), so the difference in the proportions of infected between unvaccinated and vaccinated was 10.1% (95% CI −19.5%, 61.9%). However, the difference was not significant.

## 4. Discussion

The presence of mild symptoms in the first cases did not alert those affected, who related rhinorrhea to changes in temperature and weather in Mexico City; however, once the index case was reported, it was possible to initiate diagnostic and preventive actions. 

There is permanent monitoring of environmental conditions, cleanliness, and the use of face masks in the classrooms and common areas. Nonetheless, in the (indoor) pool area, these controls are modified since there are still air extractors inside the pool area, and the contact between teachers and students is close (teachers carry the children or hold them), having a face mask as the only physical barrier. This pool area within the school, for safety reasons, has a closed-circuit camera system to monitor the activities, so the videos of the days of possible exposure were reviewed. Those who were diagnosed as cases were identified to have stopped using a face mask. 

The contagion networks allowed us to observe the presence of people with high contact with both staff and students (common contacts or brokers, D5 and A1), who, due to their functions, also have intense contact with the entire community. It is possible that the contagion with D6 may have been part of this dynamic since both identified moments of coexistence were without face mask protection (during food consumption), which coincides with D6 not having activities inside the pool area. 

It was also possible to identify potential family transmissions of students and staff (green links in Figure 3). According to the transmission network, it is possible to think that A2 presented a family contagion dynamic, given its relationship with D2. 

In the case of student K3, we assume a family transmission to his brother (K1) since K3 had contact in the pool area with a teacher who tested negative, but his brother (K1) had contact with a teacher identified as positive. 

The conditions of prevention compliance within the classrooms (environmental control, cleanliness, use of face masks, hand washing, and intentional searches) prevented the need for students to isolate, as no new cases were identified throughout the monitoring period. 

Staff members who did not work in the pool area were not affected, except in the case of A2, who was possibly infected because of a family relationship with another case (D2). 

We recognize three generations of transmission—one of teachers, followed by students, followed by family members. In the same way, we observe that the time between first exposure and the appearance of signs and symptoms of each generation is similar (48–72 h), which establishes a very short period between the exposure and the disease, from which we suppose an increase in the speed of response. Some reports indicate incubation periods similar to this one for the Delta strain [17,18]. It is worth mentioning that at the time of appearance of these cases, the number of Delta strain cases was increasing in Mexico [18]. The predominant variant in Mexico City in July 2022 was the Delta variant (93.20%), followed by the Mu variant (2.93%), and the rest for the Gamma, Alpha, and other variants. The percentages of the variants are based on the cases of Mexico City registered in the database of the diversity and distribution of variants of the SARS-CoV-2 virus of Mexico obtained by genomic sequencing [19].

In Israel, high schools fully reopened on 17 May 2020, an AR of 13.2% was recorded in the total community and an AR of 16.6% in staff members [2]. We had attack rates similar to those in Israel in students with 12.24% (95% CI 4.6–24.8), but much smaller in the total community with 6.55% (95% CI 3.9–10.0). On the other hand, in August 2021, Australian high schools reopened with limited public health mitigation measures in place, and their attack rates up to 45%; these values are very far from what we obtained with the containment measures. This study reveals how the rapid spread of COVID-19 within an educational setting was caused by the absence of public health and social measures in a low-incidence situation [6]. The AR of students was 35.3% in an elementary school classroom in California, United States of America, by the Delta variant in May 2020 as an index case to the unvaccinated teacher without a face mask despite school requirements to wear masks while indoors [4]. This outbreak showed the importance of vaccinating and wearing face masks for school staff members who are in close contact with children who are not eligible for vaccination.

Given the speed with which the number of cases expanded, and taking into account publications in this regard, we think that the early detection activities, as well as the recommendations for family isolation, facilitated quick containment of the outbreak (as soon as the index case was identified), both within the school and in the family environment of students and staff, as each generation observed a decrease in both the absolute number of cases and the proportion per affected group [2,4,8]. 

The symptomatic/asymptomatic ratio that occurred in the students is possibly associated with the viral load. In this case, one-third of the students had no symptoms [20]. In the case of teachers, practically all the detected cases developed symptoms, a relation of 12.5%. Other studies, however, have shown a 50% relation between asymptomatic people with more symptomatic people [21]. This is also possibly related to individual viral load.

The reproductive number was 4.87, considering the three primary cases and the population of the school. A value slightly below that reported for the Delta variant with a reproductive number of 5.08, which is much higher than the reproductive number of the ancestral strain with a reproductive number of 2.79 [22]. Delta infections are associated with higher viral loads and longer duration of shedding, causing higher transmissibility and reproductive number [22].

In the case of family members, they were referred directly by the parents of the students. We did not ask about the signs and symptoms of the family members; we were only informed that they were all uncomplicated cases. 

The manufacturers of the vaccines applied to the personnel reported that their main protection was to avoid severe cases and mortality, but they do not confer protection against transmission and infection, a situation that was observed in the group since the interaction between cases and contacts is where the appearance of cases can be observed; that is, the people vaccinated who had contact with the cases were the ones who fell ill, even one seriously ill and contagious to others, so individual protection measures should be continued and raises possible follow-up studies on the protection of vaccination against SARS-CoV-2 as a tool for population control.

A last point of importance was one of the teachers having been previously diagnosed with COVID-19, which reinforces the idea that a new strain was responsible for the present outbreak and raises questions about the possible immune response to infection by this agent.

### 4.1. Recommendations

Classes in the pool area were suspended until a barrier was in place to allow students and teachers to reduce transmission risk. 

The school’s prevention and control program was reinforced by means of talks and training to the staff, as well as the recommendation to consume food outdoors—maximizing the distance between diners—and requesting not to talk while without a face mask, even between vaccinated people. 

Staff were encouraged to report any at-risk contacts (e.g., immediate family members who are ill) and signs or symptoms to school health staff and facilitating activities to ensure that pupils remain active. 

A very important point was the determination that the bubbles or capsules should be kept as intact as possible; however, it was clear that there were staff who, by their internal dynamics, had a large number of contacts (brokers) and were operationally indispensable. At the same time, they pose a risk to staff and students. Therefore, despite the fact that high-efficiency masks are reserved for health personnel, and given the general availability in the market at that time, it was decided to train those who were identified as brokers in the use of high-efficiency masks and provide them with such masks for the performance of their duties while in the school’s facilities. 

We continued with intermittent staff testing and early detection actions, reinforcing prevention measures, environmental control, cleaning, and educational interventions with students regarding the implementation of preventive measures through classes led by school health staff.

### 4.2. Limitations

Possible transmissions of D6, A2, and K3 were not strictly proved. Only RT-qPCR tests were performed on those who tested positive on the rapid screening test. Signs and symptoms were self-reported. We did not evaluate the type of SARS-CoV-2 variant that the cases had. The children were not screened because they are minors, and the school cannot test the students. 

## 5. Conclusions

A student–teacher–student–family transmission sequence was identified. The area where the infection was identified was in the school swimming pool area, being an area where face masks are not worn or, in some cases, are inadequately used. 

The additional application of non-pharmaceutical prevention strategies, including screening tests and the use of face masks, is also important to protect the health of school children who are not eligible for vaccination due to their age.

In Mexico, vaccination for children between 0 and 4 years of age has not yet been approved. Vaccination in minors in Mexico is divided into two groups: in adolescents from 12 to 17 years in April 2022 and school-aged children from 5 to 11 years in November 2022. The vaccine protects minors so that they do not become seriously ill, have complications, or lose their lives, as well as cuts the chain of contagion in the community.

## Figures and Tables

**Table 1:** Distribution of attack rates by affected group and general.

Group	Cases	Population	Attack Rate	
			(%)	* 95% CI
Total Staff	8	51	15.68	7.0–28.6
Surveillance	0	7	0	0.00–49.5
Teachers/Administrative	8	35	22.85	10.4–40.1
Intendancy	0	3	0	0.00–70.7
Cleaning	0	6	0	0.00–45.9
Total Students	6	49	12.24	4.6–24.8
K1 (3 years old)	2	9	22.22	2.8–60.0
K2 (4 years old)	2	12	16.66	2.1–48.4
K3 (5 years old)	2	13	15.38	1.9–45.4
PF (6 years old)	0	10	0	0.00–30.8
1° (7 years old)	0	5	0	0.00–52.1
Family	5	190	2.63	0.8–6.0
Total Community	19	290	6.55	3.9–10.0

* CI 95%: Confidence Interval 95%.

**Table 2:** Comparison of signs and symptoms between staff members and students who were positive for SARS-CoV-2.

Signs and Symptoms	Staff Member *n* = 8	Student *n* = 6	*p*-Value *
No symptoms	1 (12.5%)	2 (33.3%)	0.538
Rhinorrhea	7 (87.5%)	4 (66.6%)	0.538
Fever	2 (25.0%)	3 (50.0%)	0.580
Cough	7 (87.5%)	0 (0.0%)	**0.005**
Anosmia	5 (62.5%)	0 (0.0%)	**0.031**
Dysgeusia	5 (62.5%)	0 (0.0%)	**0.031**
Myalgia	3 (37.5%)	0 (0.0%)	0.209
Pneumonia	1 (12.5%)	0 (0.0%)	1.000

* Fisher’s exact test. Significant *p*-values are bolded.

**Table 3:** Distribution of the vaccination of staff members and COVID-19 viral vector-based vaccines.

	Cases *n* = 8			Not Infected *n* = 43		
	Astra-Zeneca	CanSino	No vaccine	Astra-Zeneca	CanSino	No vaccine
Surveillance	1 (12.5%)	-	-	-	7 (16.3%)	-
Teachers	-	5 (62.5%)	1 (12.5%)	1 (2.3%)	17 (39.5%)	-
Administrative	-	1 (12.5%)	-	2 (4.7%)	4 (9.3%)	3 (7.0%)
Intendancy	-	-	-	-	3 (7.0%)	-
Cleaning	-	-	-	-	6 (13.9%)	-

## Data Availability

The datasets generated during and/or analyzed during the current study are available from the corresponding author upon reasonable request.
